# The HealthChain Blockchain for Electronic Health Records: Development Study

**DOI:** 10.2196/13556

**Published:** 2021-01-22

**Authors:** Yonggang Xiao, Bin Xu, Wenhao Jiang, Yunjun Wu

**Affiliations:** 1 School of Computer Science and Technology Hubei University of Science and Technology Xianning China; 2 Chongqing Aerospace Polytechnic Chongqing China; 3 School of Computer Science and Technology Chongqing University of Posts and Telecommunications Chongqing China

**Keywords:** electronic health record, distributed ledger, privacy preservation, proof of authority, chaincode application programming interface

## Abstract

**Background:**

Health care professionals are required to maintain accurate health records of patients. Furthermore, these records should be shared across different health care organizations for professionals to have a complete review of medical history and avoid missing important information. Nowadays, health care providers use electronic health records (EHRs) as a key to the implementation of these goals and delivery of quality care. However, there are technical and legal hurdles that prevent the adoption of these systems, such as concerns about performance and privacy issues.

**Objective:**

This study aimed to build and evaluate an experimental blockchain for EHRs, named HealthChain, which overcomes the disadvantages of traditional EHR systems.

**Methods:**

HealthChain is built based on consortium blockchain technology. Specifically, three organizations, namely hospitals, insurance providers, and governmental agencies, form a consortium that operates under a governance model, which enforces the business logic agreed by all participants. Every peer node hosts an instance of the distributed ledger consisting of EHRs and an instance of chaincode regulating the permissions of participants. Designated orderers establish consensus on the order of EHRs and then disseminate blocks to peers.

**Results:**

HealthChain achieves functional and nonfunctional requirements. It can store EHRs in a distributed ledger and share them among different participants. Moreover, it demonstrates superior features, such as privacy preservation, security, and high throughput. These are the main reasons why HealthChain is proposed.

**Conclusions:**

Consortium blockchain technology can help to build new EHR systems and solve the problems that prevent the adoption of traditional systems.

## Introduction

It has long been believed that electronic health records (EHRs) should be maintained across time and space, and could be accessed at any time and any place within the law [1]. In the first stage of digitization, we store a patient’s medical history within the jurisdiction of a health care provider irrespective whether electronic medical records (EMRs) are on a local server [2] or in the cloud [3]. Such EMR systems have no essential difference with old-fashioned paper-based ones, since information technology just takes the management of medical records from paper folders to hard drives. EMRs do not travel out of a practice [4], and they make their way to other practices by faxes or signed documents, which is time consuming.

In the second stage, authorized doctors and staff create, manage, and consult EHRs across more than one health care organization, allowing interoperability between disparate EHR systems [5]. That is, EHRs possess the ability to share medical information among health care providers and follow a patient’s information across multiple health care organizations [6]. In the United States, EHR exchange involves a common platform, the Nationwide Health Information Network, which is a set of standards, services, and policies that enable secure health information exchange over the internet [7].

However, there are technical and legal hurdles that prevent the adoption of these systems. First, these systems perform poorly in terms of data availability, data integrity, and retrieval rate when EHRs are stored under a distributed or institution-centric model [8]. Second, people always worry about the issues of privacy and data breaches [9] when EHRs are beyond their control, even if health care providers and governmental agencies claim that these systems are Health Insurance Portability and Accountability Act (HIPAA) compliant [10]. After all, 11,581,616,452 records have been breached since 2005, and this has been reported through either government agencies or verifiable media sources [11]. Third, patient-reported data do not always get recorded in a patient’s EHRs since doctor-patient communication is not always possible, which impacts the quality of care [12]. Therefore, a kind of patient-reporting mechanism is needed for precision medicine [13].

In this paper, HealthChain is proposed to address the above-mentioned issues. It is a blockchain [14-16] for EHRs, that is, a growing list of blocks that consist of records and are linked using a cryptographic hash [17]. The blockchain has several advantages. First, the blockchain is a distributed peer-to-peer database where data availability, data integrity, and response time are guaranteed [18,19]. Blockchains can facilitate Internet of Things security in eHealth [20]. Second, the blockchain operates under a governance model, which enforces the business logic agreed by all participants. Therefore, we can exploit a smart contract or chaincode to regulate the access control policy [21-23] and achieve HIPAA compliance. Third, the blockchain is managed collectively by its stakeholders, some of whom have the right to record data in the block that cannot be altered retroactively [24]. In our design, even patients can report personal health records (PHRs) [5] on the ledger. From our perspective, applying blockchain technology to EHR systems denotes the advent of the third stage of digitization.

Specifically, the proposed HealthChain is different from other EHR blockchains owing to the following features. First, HealthChain is a consortium blockchain [25]. Multiple organizations, namely hospitals, insurance providers, and governmental agencies, come together to form the consortium. The business logic is determined by the governance model that is agreed by the consortium at the beginning, rather than the trustless model of other medical blockchains [26-28]. Second, HealthChain performs well in the following aspects: data availability, data integrity, and retrieval success rate, that is, HealthChain is always online even if a few servers crash. Genuine EHRs are stored since they are signed by valid stakeholders. We can successfully access the ledger anytime because of load balancing. Third, HealthChain uses proof of authority (PoA) as its consensus protocol. Designated, authenticated, and trustworthy orderers are responsible for generating valid blocks, that is, as long as the blocks are signed by one of these orderers, they are accepted by all participants. PoA is different from other consensus protocols, such as proof of work [21,29,30] and Practical Byzantine Fault Tolerance (PBFT) [31-33]. Fourth, different users possess different chaincode application programming interfaces (APIs) in HealthChain, which is specified by the governance model. Therefore, we can define the way that users interact with the ledger and achieve the access control policy. Fifth, HealthChain performs well in the experimental environment. This paper uses the following four metrics to evaluate performance: read latency, read throughput, transaction latency, and transaction throughput [34].

In this paper, we build a consortium blockchain for EHRs, named HealthChain, which has advantages over traditional EHR systems and other medical blockchains recently proposed. Moreover, we evaluate the performance of HealthChain through blockchain-specific metrics.

## Methods

### Diagram of HealthChain

**Figure 1:**
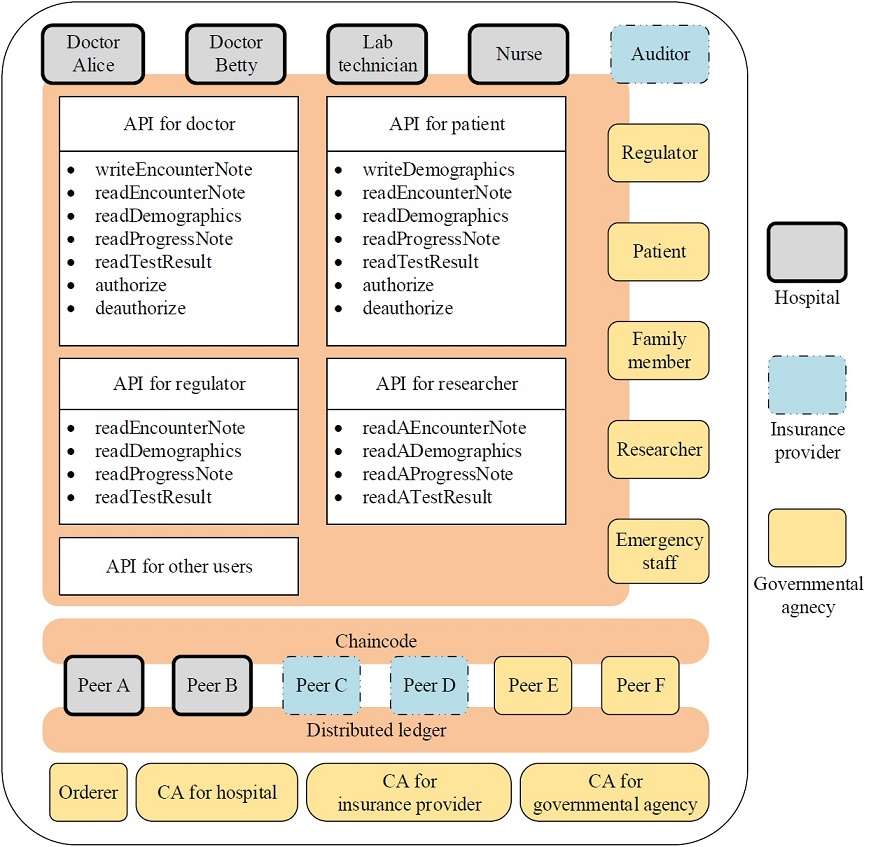
The diagram of HealthChain. Three kinds of organizations contribute nodes to the blockchain network. API: application programming interface; CA: certificate authority.

The consortium consists of the following three kinds of organizations: hospitals, insurance providers, and governmental agencies. Our experimental HealthChain comprises two of each type, as shown in Figure 1. Peers A and B are servers owned by two respective hospitals, and they serve client applications of the following three kinds of users: doctors (such as Alice and Betty), lab technicians, and nurses. Peers C and D are servers contributed by two respective insurance providers, and they serve clients for auditors. Peers E and F are servers belonging to two respective governmental agencies, and they serve client applications of the following five kinds of users: regulators, patients, family members, researchers, and emergency staff. Besides, governmental agencies contribute one orderer and three certificate authorities (CAs) for three kinds of organizations. Note that these servers can reside in the cloud, in the data center of the organizations, or on a single machine.

Although there is no centralized node in the HealthChain network, the organizations come together under the governance model, which regulates the behavior of all actors such as peers and users. The governance model reflects the business logic of health care in real scenarios. It specifies the way that users interact with the ledger, and enables the privacy and confidentiality of EHRs. HealthChain implements the governance model via the combination of a membership service provider (MSP), chaincode, and consensus protocol. These components are explained below, and we introduce the roles of all nodes constituting the network.

CAs are trusted authorities, generating certificates and key material for actors. The MSP is implemented by these CAs, as the generated certificates can provide information about valid identities for an organization. Moreover, all actors in HealthChain can be verified by each other, and the MSP helps achieve fine-grained access and trace behavior of actors.

Peers make up the physical structure of our network. The distributed ledger and chaincode are shared among them, as illustrated in Figure 1. They are both endorsing and committing peers in HealthChain. As endorsing peers, they are designated by the consortium to execute chaincode in simulation. The chaincode implements APIs, which are divided into different groups granted to different users [32], that is, the chaincode specifies who has what access permissions to which part of the ledger and implements the fine-grained access required by law [1]. In Figure 1, we list APIs for doctors, patients, regulators, and researchers.

As committing peers, they validate blocks and commit them to their copies of the ledger. Most importantly, they check the identity of who executes the EHR request and the identity of who packages EHR transactions into a block. This is why the consensus protocol adopted in HealthChain is called PoA.

Orderers are trusted by all organizations, and they are responsible for ordering EHRs into a block on a first-come-first-serve basis. The signature of the block writer (ie, the orderer) is contained in the block. Prior to the commitment, the peers must make sure that the signature comes from an authenticated and authorized orderer, that is, the validity of a block depends on the identity of the orderer.

### Identities and Anonymization

CAs dispense X.509 certificates to identify servers and clients. X.509 certificates are used in the lifecycle of transactions. For example, the EHR request contains the certificate of a client. Meanwhile, the EHR response includes the certificate of an endorsing server. When enforcing the access control of HealthChain, the chaincode extracts the certificate from the transaction request, acquires the identity of the client, and queries whether the access is authorized.

Considering privacy preservation, the identities of patients and health care providers need to be anonymized when researchers access the ledger via the APIs for them. During the anonymization, we use the hash of identities instead of the identities themselves. Thus, the identities are kept private, but the relationship between patients and health care providers is retained. Specifically, SHA256 is adopted as it is the default hash algorithm in Hyperledger Fabric. The hash of the patient ID combined with the timestamp is calculated. The purpose of introducing the timestamp is to prevent getting the same hash among queries.

### Access Rights of Users

**Figure 2:**
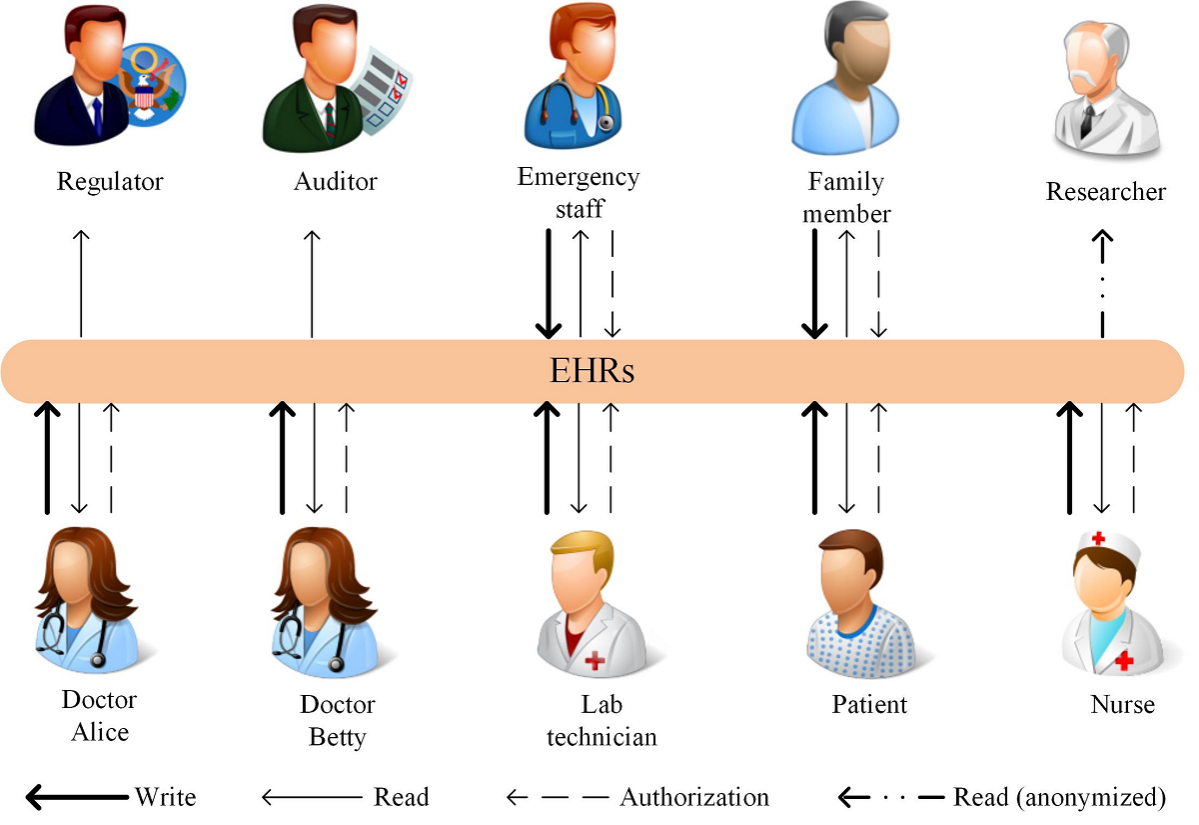
The access rights of users in HealthChain. Different users have different access qualifications for electronic health records (EHRs).

HealthChain is a distributed and append-only ledger shared among many users with different access rights. To achieve a delicate balance between privacy and availability, fine-grained access to the ledger is implemented. Figure 2 illustrates the following access rights of users in HealthChain: write permission, read permission, authorization permission, and read permission with EHRs anonymized.

Patients manage and control access to the PHRs, conceptually including patient-reported information and EHRs. The former includes various contents, such as demographics, allergies, and monitoring data collected from instruments [5]. The latter refers to medical records updated by doctors and staff. Patients can authorize their family members or health care providers to write and read their health information [35], reducing the risk of data replication and tracking possible trends and changes in their health.

Doctors, nurses, emergency staff, and lab technicians manage and control access to the EHRs updated by themselves. Furthermore, they can use or disclose protected health information for treatment, payment, or health care operations without patients’ authorization [36]. Therefore, they have authorization permission to grant write or read permission to other covered entities, whereby the EHRs are shared across health care organizations.

Regulators and insurance providers only have read permission to the ledger. Regulators, such as the Department of Health & Human Services, ensure that the business logic of HealthChain has been respected well, and all participants behave appropriately. When there is a dispute, they can make decisions based on the ledger, which is tamper-resistant and unforgeable [37]. Insurance providers process medical claims and evaluate their validity according to the records on HealthChain.

Researchers are engaged in public health activities such as disease surveillance. HealthChain can provide trustworthy data for this purpose because of the transparency of the data aggregation process [32]. However, the data on the ledger should be anonymized before being used for privacy preservation.

### Implementation of the Authorization

**Figure 3:**
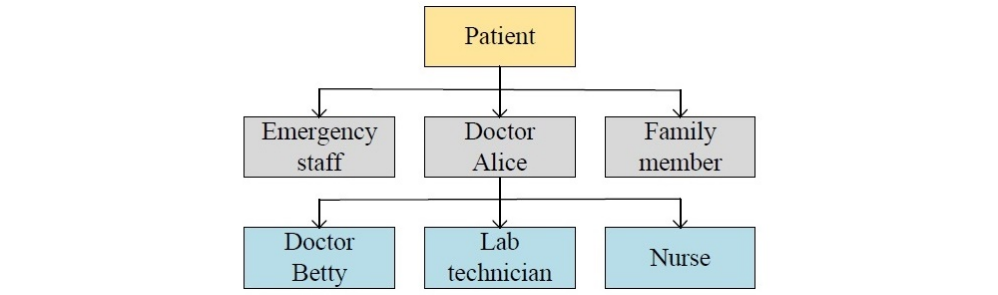
The authorization hierarchy in HealthChain. The arrows denote the authorization operation.

The authorization is represented by a tree data structure, as shown in Figure 3. All access rights are authorized from the patient (ie, the root of the tree). Parent nodes grant child nodes permissions such as read, write, and authorization. For example, the patient grants all three permissions to doctor Alice, and doctor Alice grants all three permissions to doctor Betty, and so on. Besides, the other three roles (ie, regulator, auditor, and researcher) have only read access to the ledger. While the tree data structure is the logical design of the authorization, the records or transactions on the ledger are its physical implementation.

**Figure 4:**
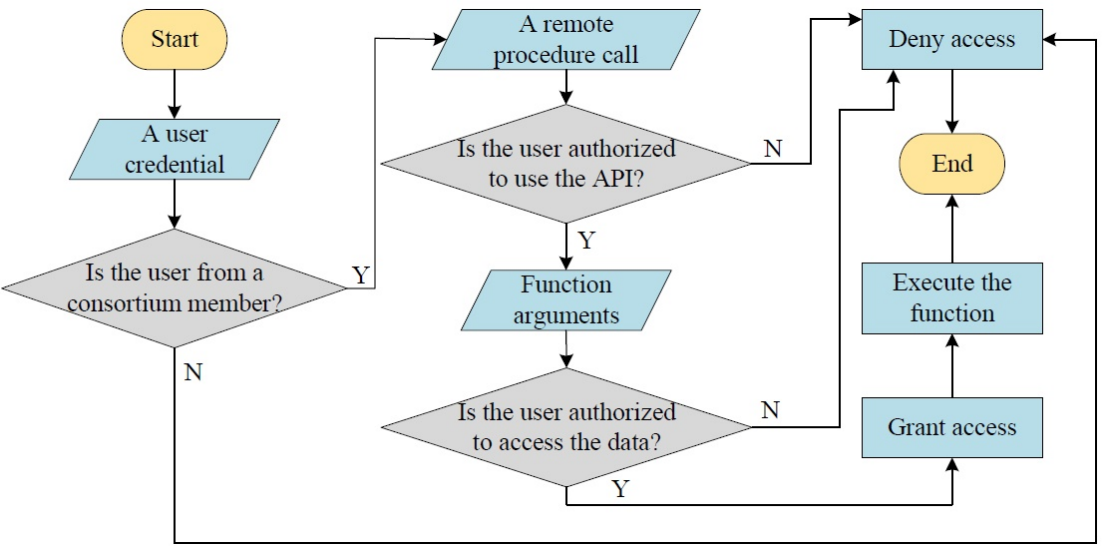
The fine-grained access control implemented by the chaincode. N: no; Y: yes.

The algorithm addressing authentication and authorization is presented in Figure 4, and it is implemented by the chaincode. The key parts are the three decision symbols, representing the prerequisites for the success of transactions. First, only consortium members can access the ledger; others have no right at all. Second, different types of users have different access permissions for chaincode APIs, even in the same consortium. Third, a user cannot access the data of another if the former is not authorized by the latter. For example, if a person named Carl would like to submit an encounter note about a patient named Steve, he needs to satisfy the following three conditions: he is from a hospital within the consortium, he is a doctor, and he has authorization from Steve.

### Types and Lifecycle of EHRs

**Figure 5:**
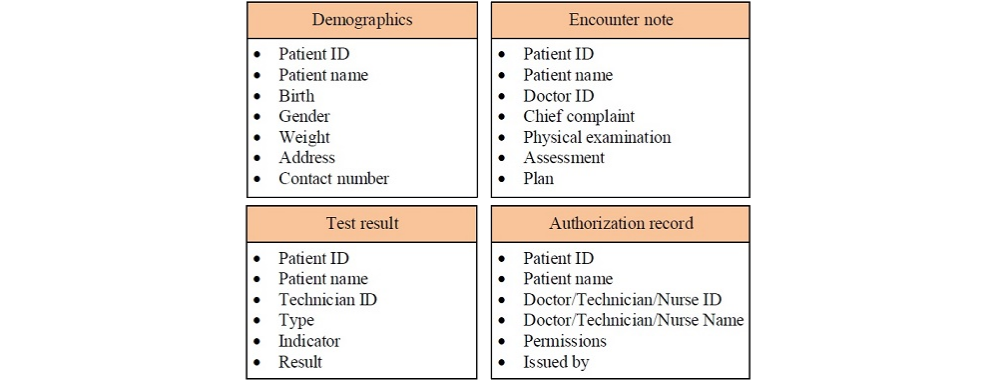
The types of electronic health records. Only four examples are listed here.

There are different kinds of EHRs submitted by different users. For the sake of brevity, Figure 5 lists only four of them, illustrating the data structures of the demographics, encounter note, test result, and authorization record. For example, the encounter note includes the following fields: patient ID, patient name, doctor ID, chief complaint, physical examination, assessment, and plan.

**Figure 6:**
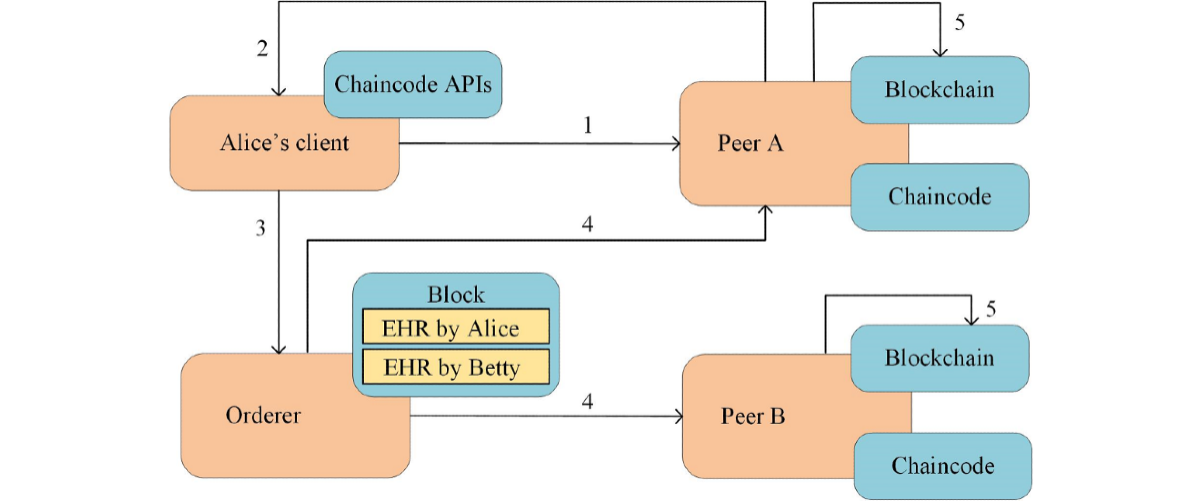
The lifecycle of electronic health records (EHRs). They start with the request from a client and end with the commitment on all peers. API: application programming interface.

Every EHR corresponds to a transaction, which needs to be executed and eventually included in the ledger. All EHRs have the same lifecycle [16], which is illustrated in Figure 6 and is explained as follows:

1. When Doctor Alice needs to record an encounter note on the ledger, her client application sends an EHR request to the endorsing peer. The request is formatted as a remote procedure call through chaincode APIs [38].
2. The endorsing peer checks the validity of the incoming request (the format, the signature, and the access permission). Thereafter, the request is processed by the chaincode, which outputs an EHR response, including returned value, read set, and write set.
3. Alice’s client checks the signature of the incoming response, assembles the response and the signature into an EHR transaction, and sends the transaction to an orderer.
4. The orderer simply receives transactions from all clients (including Alice’s client), orders them chronologically, and creates a block of transactions. Thereafter, the orderer delivers the block to all peers in the network.
5. Every peer checks the validity of the incoming block, including the signature and the version number. After that, the block is committed to the ledger and so is the EHR submitted by Alice.

### Structure of the Ledger

**Figure 7:**
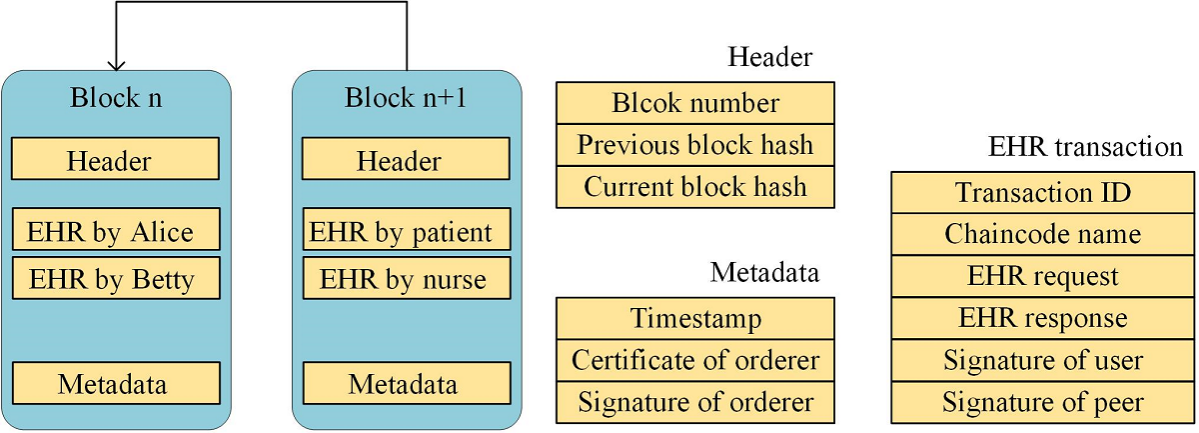
The structure of the transaction, the block, and the ledger. EHR: electronic health record.

The ledger is the database of EHRs across time and space. As shown in Figure 7, it is structured as interlinked blocks, each of which contains chronologically ordered EHR transactions.

A block consists of the following three sections: header, EHR transactions, and metadata. The header comprises the block number, the previous block hash, and the current block hash. The EHR transactions are submitted by users such as doctors and patients. The metadata comprises the timestamp, as well as the certificate and signature of an orderer. Besides, an EHR transaction consists of the following six fields: the transaction ID, the chaincode name, the EHR request, the EHR response, and the signatures of the user and peer.

The previous block hash of block n+1 is equal to the current block hash of block n, such that the blocks are interlinked; hence, the name blockchain. The biggest difference between HealthChain and other blockchains is that blocks do not need to contain a nonce field to achieve the given pattern of cryptographic hash values. The validity of the block is only dependent on PoA, instead of proof of work. Thus, HealthChain can achieve low transaction latency.

## Results

### Experimental Environment

The prototype of HealthChain is implemented with Hyperledger Fabric v1.4.1, an enterprise-grade permissioned distributed ledger platform [16]. It is deployed on a machine with an Intel Xeon E5-26xx v4 2.4 GHz CPU and 2 GB RAM running Ubuntu 16.04.1 LTS. All servers are built with Docker 18.06.1-ce, that is, all peers and orderers are virtualized into containers sharing the hardware and the operating system kernel [39]. The HealthChain network is created by Docker Compose, a tool for defining and running multicontainer Docker applications [40].

Besides, the two parameters of batch timeout and batch size have a high impact on the performance of HealthChain. The former denotes the maximum time to wait before creating a block, and the latter is the maximum number of EHR transactions in a block. No matter which one is satisfied first, the block is generated. This paper tests the performance of HealthChain with the parameters varying. We ran every transaction three times, and the average values of latency and throughput are provided in the paper.

### Example of the Working of HealthChain

In this section, we present an example to illustrate how HealthChain works. Note that all tasks are completed through our chaincode APIs described in Figure 1. The medical data are extracted from a previous report [41].

First, the patient client of Steve Apple prepares personal information. Consequently, the following demographic record is committed to the ledger.

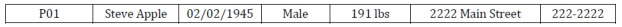



Second, when Steve decides to see a doctor named Carl Savem, he submits an authorization record to grant permissions for health care. Thus, the doctor is able to perform read, write, and even authorization operations on the ledger.

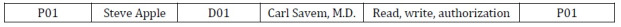



Third, after knowing Steve’s feelings about the health state and doing a medical examination, Carl enters the diagnosis and the instruction for treatment.

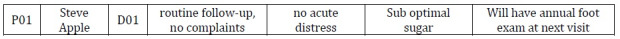



Fourth, to check whether there is a change in the lipid profile, Carl also writes an authorization record to order a test provided by a technician named John Doe.

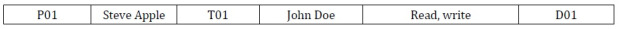



Finally, John Doe puts the following report on the ledger.

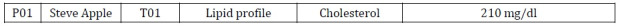



Besides, when researchers read the above encounter note, the anonymization scheme in HealthChain takes effect, resulting in the following record. The first three items are the hashes of corresponding items in the original record and timestamps.

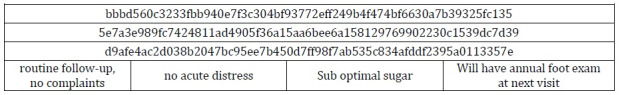



### Security Test

We test the security of HealthChain from three aspects. First, a patient tries to access the ledger on an unavailable server, peer E. The below command queries the encounter notes about the patient with ID equal to P01, but it fails due to the unavailability of peer E, whose domain name is peer0.org3.health.com. After peer0.org3.health.com is replaced with peer0.org1.health.com, that is, the domain name of peer A, the command runs successfully since peer A is still available.

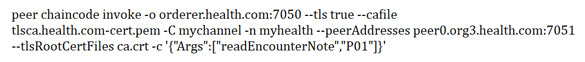



Second, an invalid user tries to access the ledger. However, this operation is immediately denied by the system since the identity cannot be authenticated. After we delete the digital certificate of Steve Apple, the above command fails, and the below information is returned.

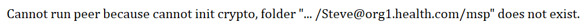



Third, a doctor tries to tamper with an EHR. However, the history of the EHR is recorded on the ledger. We can query the history of the encounter note about P01. It is easy to figure out that “physicalExamination” has changed from “no acute distress” to “acute distress.”

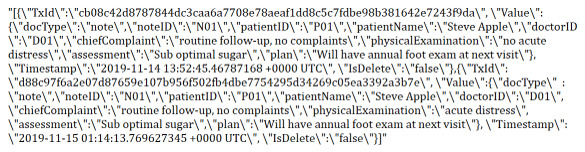



In conclusion, HealthChain performs well in several aspects, namely data availability, access control, and data integrity.

### Test Scenario: A Single Read

The read operation refers to retrieving or querying EHRs, and there is no change to the ledger. Users may read the EHRs submitted by themselves or others. We can give some use cases here. Doctors query the demographics of a patient or the diagnoses from former doctors. Patients read medical notes from doctors or lab results from lab technicians [24]. Insurance providers inspect EHRs to validate the necessity of them. Read latency is the time between when the EHR read request is submitted and when the EHR response is returned, corresponding to steps 1 and 2 in Figure 6.

Table 1 shows the example data of a single read operation under different parameter conditions. It can be seen that HealthChain has the read latency of about 0.1 s irrespective of the parameters. For example, the first row shows the process of a read operation when Doctor Alice retrieves an EHR from peer A. She sends an EHR request at 0 s, peer A finishes retrieving at 0.136 s, and an EHR response is returned to her at 0.142 s. Therefore, the read latency of 0.142 s is obtained with batch timeout equal to 20 s and batch size equal to 1000. The two parameters are irrelevant since there is no block generated during the read process.

**Table 1:** Example read latency data at different nodes.

Number	Batch timeout (s)	Batch size (n)	Client	Sever	Read request (s)	Read (s)	Response (s)	Read latency (s)
1	20	1000	Doctor	Peer A	0	0.136	0.142	0.142
2	20	1	Insurance provider	Peer C	0	0.117	0.122	0.122
3	2	10	Patient	Peer E	0	0.099	0.105	0.105

### Test Scenario: Concurrent Reads

Usually, many users read from the ledger simultaneously and the read operations are executed in overlapping time periods. For example, doctors, patients, and insurance providers may read EHRs from the ledger concurrently in accordance with their needs. Read throughput is obtained by the number of read operations completed in a specific time period, indicated as reads per second (RPS).

Table 2 shows the example data of 1000 concurrent reads under different parameter conditions. It can be observed that every server of HealthChain has a read throughput of around 10 RPS in spite of varying parameters. For example, the first row shows the process of 1000 concurrent read operations when users try to retrieve EHRs from peer A at the same time. The first EHR request happens at 0 s, peer A finishes the 1000th read at 97.513 s, and the 1000th response happens at 97.521 s. Therefore, the read throughput reaches 10.259 RPS with batch timeout of 20 s and batch size of 1000. The two parameters are irrelevant as no block is written on the ledger. Furthermore, the read throughput of the whole network is the summation of the throughput of all peers as the read operations on one peer are independent of those on another.

**Table 2:** Example read throughput data at different nodes.

Number	Batch timeout (s)	Batch size (n)	Sever	1st read Request (s)	1000th read (s)	1000th response (s)	Read latency (s)	Read throughput (reads per s)
1	20	1000	Peer A	0	97.513	97.521	97.521	10.259
2	20	1	Peer C	0	95.847	95.855	95.855	10.440
3	2	10	Peer E	0	98.871	98.876	98.876	10.115

### Test Scenario: A Single Write

The write operation refers to submitting EHRs to the ledger, and there are changes involved. Users may create and submit EHRs regarding a patient. We can list some use cases here. Doctors create encounter notes after meeting with patients or progress notes during the course of a hospitalization. Patients update demographics or report their clinical status [22]. Lab technicians report test results. Transaction latency is the time between when an EHR write request is submitted and when the EHR transaction is widely available in the network, corresponding to all five steps in Figure 6.

Table 3 shows the example data of a single write operation with different parameter conditions. It can be seen that the transaction latency depends on the two parameters. For example, the first row shows the process of a write operation when doctor Alice submits an EHR to the ledger. She sends an EHR request at 0 s, peer A endorses the EHR at 0.118 s, and the orderer generates a block containing the EHR transaction at 20.166 s. Subsequently, six peers separately commit the block to the ledger at 20.370 s, 20.352 s, 20.379 s, 20.379 s, 20.363 s, and 20.379 s. Therefore, the EHR transaction is available on all peers at the latest time, namely 20.379 s. Consequently, the transaction latency of 20.379 s is obtained with batch timeout of 20 s and batch size of 1000.

**Table 3:** Example transaction latency data at different nodes.

No.	Batch timeout (s)	Batch size (n)	Client	Write request (s)	Endorsement on A (s)	Write on orderer (s)	Commitment on A (s)	Commitment on B (s)	Commitment on C (s)	Commitment on D (s)	Commitment on E (s)	Commitment on F (s)	Latest (s)	Transaction latency (s)
1	20	1000	Doctor	0	0.118	20.166	20.370	20.352	20.379	20.379	20.363	20.379	20.379	20.379
2	20	1	Patient	0	0.110	0.163	0.434	0.424	0.423	0.428	0.437	0.418	0.437	0.437
3	2	10	Lab technician	0	0.099	2.141	2.292	2.306	2.290	2.305	2.306	2.291	2.306	2.306

We explain the transaction latency in Table 3. In the first and third cases, the orderer has to wait for 20 s and 2 s, respectively, before creating a block because there is only one incoming EHR transaction that needs to be packaged into the block and the batch timeout occurs first. In the second case, the batch size is 1 and there happens to be one EHR transaction, so the batch size is satisfied first, and the orderer does not have to wait for 20 s before creating the block.

### Test Scenario: Concurrent Writes

Usually, many users write to the ledger simultaneously and the write operations are executed in overlapping time periods. For example, doctors, patients, and lab technicians may write EHRs to the ledger concurrently as needed. Transaction throughput is calculated by the number of EHR transactions committed by the network in a specific time period, expressed as transactions per second (TPS).

Table 4 shows the example data of 1000 concurrent writes with different parameter conditions. It can be seen that the transaction throughput is determined by the two parameters. For example, the first row shows the process of 1000 concurrent write operations when users try to submit EHRs to the ledger at the same time. The first EHR request occurs at 0 s, peer A finishes the 1000th endorsement at 132.224 s, and the orderer generates the last block at 139.930 s. Subsequently, six peers of the network separately commit the last block at 141.498 s, 141.936 s, 141.933 s, 141.400 s, 141.042 s, and 141.976 s. Therefore, the 1000 EHR transactions are available on all peers at the latest time, namely 141.976 s. Consequently, the transaction throughput reaches 7.043 TPS with batch timeout of 20 s and batch size of 1000.

**Table 4:** Example transaction throughput data at different nodes.

Number	Batch timeout (s)	Batch size (n)	1st write request (s)	1000th endorsement on A (s)	Last write on orderer (s)	Last commitment on A (s)	Last commitment on B (s)	Last commitment on C (s)	Last commitment on D (s)	Last commitment on E (s)	Last commitment on F (s)	Latest (s)	Transaction latency (s)	Transaction throughput (TPS)
1	20	1000	0	132.224	139.930	141.498	141.936	141.933	141.400	141.042	141.976	141.976	141.976	7.043
2	20	1	0	241.035	241.519	633.266	608.020	635.720	608.755	636.811	609.583	636.811	636.811	1.570
3	2	10	0	157.787	157.818	158.096	158.202	158.153	158.100	158.094	158.126	158.202	158.202	6.321

We can account for the transaction throughput in Table 4. In the first case, blocks are created every 20 s. Because it takes more than 20 s for 1000 transactions to come, the batch timeout happens first. In the second case, blocks are created every EHR transaction since the batch size is 1. In the third case, blocks are created every 10 EHR transactions. Because it takes less than 2 s for 10 transactions to come, the batch size happens first. It can be observed that the transaction throughput is inversely proportional to the number of blocks generated. Generating more blocks increases the likelihood of network congestion caused by the gossip protocol [16].

## Discussion

### Features of HealthChain

Based on the content that we have covered, the features of HealthChain are summarized as follows. First, HealthChain is permissioned. Unlike with a public permissionless network, all users like doctors and patients are certificated by the MSP and therefore are identifiable to each other, rather than anonymous and fully untrusted. Unauthorized or unknown users are not allowed to access the ledger. Second, HealthChain is immutable, that is, once EHRs have been added to the chain, they cannot be changed. This append-only property depends on the fact that the blocks are interlinked via hash references. Thus, HealthChain is the authoritative source of patients’ treatment history. Third, HealthChain is transparent. Health care stakeholders come together to constitute the blockchain network, and none of them controls the whole system. Every operation initiated by users is checked against the governance model, which regulates and monitors the behavior of all actors. Furthermore, EHRs like encounter notes and lab results are shared among covered users, who know what is going on during the course of treatment. Fourth, HealthChain is HIPAA compliant with the privacy rule and security rule. Privacy policies are implemented through chaincode. The use of patient information is denied without authorization. Patients have final control over the EHRs, and they can grant write and read permissions to other covered entities. Besides, HealthChain exploits the Transport Layer Security (TLS) protocol to provide communication security over the network [16]. Fifth, HealthChain is scalable. Not all peers are involved in the transaction execution, and not all orderers are involved in the block generation. Therefore, parallel transaction execution and block generation are allowed, and HealthChain can easily support more nodes, though there are only six peers and one orderer in our experimental environment. Sixth, HealthChain has good performance. Even in our experimental environment, the read latency was about 0.1 s, and the read throughput of every peer was about 10 RPS. HealthChain achieves a transaction latency of about 0.4 s with batch size of 1, and it supports a transaction throughput of about 7 TPS with batch timeout of 20 s and batch size of 1000. In contrast, Bitcoin and Ethereum take 600 s and 10 s, respectively, to write a transaction on the ledger [42].

### Parameter Setting

Performance of HealthChain is affected by many variables such as network size and limits of the hardware. Here, we discuss two parameters that we configure in the experiments, that is, batch timeout and batch size.

To achieve good performance, we should adjust batch timeout considering the permitted maximum transaction latency and set the batch size according to the rate indicating how many EHR transactions are submitted to the orderer during a specific period. The two parameters should be separately proportional to the permitted latency and the rate. As shown in the experiments, HealthChain obtains the lowest transaction latency when there is only one EHR transaction and the batch size is set to 1. It achieves the highest transaction throughput when there are 1000 transactions and the batch size is 1000.

### Limitations

The prototype of HealthChain has two disadvantages. First, the only orderer causes a single point of failure. If the orderer fails, EHR transactions cannot be ordered into a block, causing failure of the entire system. To address this problem, we can deploy an ordering service consisting of a set of ordering service nodes and a Kafka cluster with its ZooKeeper ensemble [16]. This will help to not only build a crash fault-tolerant system but also increase the performance owing to load balancing.

Second, the read latency increases with the growth of the ledger. The ledger is implemented as a file on the disk considering the append-only write operation. At the same time, the read operation is also common in HealthChain. However, EHRs may scatter over the file with time, resulting in difficulty in searching for them. To solve the problem, we can create an index of patients on the ledger, making the read operation fast.

### Conclusions

In this study, we built and evaluated HealthChain, which is an EHR consortium blockchain that operates under a governance model. It ensures data availability and data integrity. It provides chaincode APIs to accommodate the requirements from different clients and implements fine-grained access control. Besides, a way to anonymize EHRs is introduced. HealthChain adopts PoA as its consensus algorithm. The functionality of HealthChain was observed in the experiments. We described the performance of the system through latency and throughput.

## Abbreviations

API: application programming interface
CA: certificate authority
EHR: electronic health record
EMR: electronic medical record
HIPAA: Health Insurance Portability and Accountability Act
MSP: membership service provider
PBFT: Practical Byzantine Fault Tolerance
PHR: personal health record
PoA: proof of authority
RPS: reads per second
TPS: transactions per second
